# Discussion of Heated Tobacco Products on Twitter Following IQOS’s Modified-Risk Tobacco Product Authorization and US Import Ban: Content Analysis

**DOI:** 10.2196/53938

**Published:** 2024-10-24

**Authors:** Minji Kim, Julia Vassey, Dongmei Li, Artur Galimov, Eileen Han, Matthew G Kirkpatrick, Cassandra A Stanton, Jenny E Ozga, Sarah Lee, Jennifer B Unger

**Affiliations:** 1 Department of Health Promotion, Education, and Behavior Arnold School of Public Health University of South Carolina Columbia, SC United States; 2 Department of Population and Public Health Sciences Keck School of Medicine University of Southern California Los Angeles, CA United States; 3 Department of Clinical and Translational Research University of Rochester Medical Center Rochester, NY United States; 4 Center for Tobacco Control Research and Education University of California, San Francisco San Francisco, CA United States; 5 Department of Psychology University of Southern California Los Angeles, CA United States; 6 Behavioral Health and Health Policy Practice Westat Rockville, MD United States

**Keywords:** heated tobacco products, IQOS, social media, Twitter, tobacco control, modified-risk tobacco product authorization, MRTP authorization, tobacco regulatory science, import ban, observational study, public opinion, content analysis

## Abstract

**Background:**

Understanding public opinions about emerging tobacco products is important to inform future interventions and regulatory decisions. Heated tobacco products (HTPs) are an emerging tobacco product category promoted by the tobacco industry as a “better alternative” to combustible cigarettes. Philip Morris International’s IQOS is leading the global HTP market and recently has been subject to important policy events, including the US Food and Drug Administration’s (FDA) modified-risk tobacco product (MRTP) authorization (July 2020) and the US import ban (November 2021). Although limited in their legal implications outside the United States, these policy events have been quoted in global news outlets and Philip Morris International’s promotional communications, showing how they may potentially impact global tobacco regulation. Given the impending return of IQOS to the US market, understanding how the policy events were received through social media discourse will provide valuable insights to inform global tobacco control policy.

**Objective:**

This study aims to examine HTP-related social media discourse around important policy events.

**Methods:**

We analyzed HTP-related posts on Twitter during the time period that included IQOS’s MRTP authorization in the United States and the US import ban, examining personal testimonial, news/information, and direct marketing/retail tweets separately. We also examined how the tweets discussed health and policy. A total of 10,454 public English tweets (posted from June 2020 to December 2021) were collected using HTP-related keywords. We randomly sampled 2796 (26.7%) tweets and conducted a content analysis. We used pairwise co-occurrence analyses to evaluate connections across themes.

**Results:**

Tweet volumes peaked around IQOS-related policy events. Among all tweets, personal testimonials were the most common (1613/2796, 57.7%), followed by news/information (862/2796, 30.8%) and direct marketing/retail (321/2796, 11%). Among personal testimonials, more tweets were positive (495/1613, 30.7%) than negative (372/1613, 23.1%), often comparing the health risks of HTPs with cigarettes (402/1613, 24.9%) or vaping products (252/1613, 15.6%). Approximately 10% (31/321) of the direct marketing/retail tweets promoted international delivery, suggesting cross-border promotion. More than a quarter of tweets (809/2796, 28.9%) discussed US and global policy, including misinterpretation about IQOS being a “safer” tobacco product after the US FDA’s MRTP authorization. Neutral testimonials mentioning the IQOS brand (634/1613, 39.3%) and discussing policy (378/1613, 23.4%) showed the largest pairwise co-occurrence.

**Conclusions:**

Results suggest the need for careful communication about the meaning of MRTP authorizations and relative risks of tobacco products. Many tweets expressed HTP-favorable opinions referring to reduced health risks, even though the US FDA has denied marketing of the HTP with reduced risk claims. The popularity of social media as an information source with global reach poses unique challenges in health communication and health policies. While many countries restrict tobacco marketing via the web, our results suggest that retailers may circumvent such regulations by operating overseas.

## Introduction

Heated tobacco products (HTPs) use electronic heating devices that heat processed tobacco sticks to create nicotine-containing aerosol [1-3]. Philip Morris International (PMI) introduced IQOS in Switzerland and Japan in 2014 and has been leading the global HTP market. Other popular products in the category include British American Tobacco’s (BAT) Glo, Japan Tobacco International’s Ploom, and KT&G’s Lil [4].

IQOS launched in selected markets in the United States in 2019, after its premarket tobacco product authorization by the US Food and Drug Administration (FDA) in 2019 [5,6]. In July 2020, the FDA authorized IQOS’s modified risk tobacco product (MRTP) claims that complete switching to IQOS from cigarettes would reduce exposure to harmful and potentially harmful chemicals (“reduced exposure” claims), while denying authorization marketing claims that using IQOS can reduce the risks of tobacco-related diseases [7]. Also in 2020, two major patent infringement lawsuits were filed against PMI by BAT (April 2020) and Healthier Choices Management Corp (November 2020), with hearings at the US International Trade Commission (ITC) starting in the end of January 2021 [8]. As a result, in May 2021, PMI lost the first round of BAT lawsuits. In September 2021, the US ITC banned the import of IQOS into the United States, which took effect in November 2021 [9]. As of March 2024, IQOS is still not available in the United States, although PMI recently reached an agreement with BAT to resolve all ongoing patent infringement litigation [10], which will result in resuming sales of IQOS in the United States.

Even before the US MRTP authorization, HTPs have been promoted as “cleaner,” “high-tech,” and “better” alternatives to combustible tobacco products in other countries such as Japan [11] and South Korea [3], using explicit and implicit claims, which were often interpreted as HTPs being “less harmful” than cigarettes [12,13]. The tobacco industry has argued that HTPs are different from other tobacco products, primarily by claiming that they pose a lower level of health risk, in an effort to avoid stricter regulation [14]. However, earlier research suggests that people who smoke cigarettes who adopted HTPs continued smoking [15], potentially leading to greater nicotine exposure and addiction by using multiple tobacco products.

The effects of promotional efforts, policy events, and media coverage on public opinion about emerging tobacco products, including HTPs, need further research. Although the MRTP authorization applies only to the United States, this decision was widely quoted by global news media, often including inaccurate and misleading information about IQOS being “safer” than cigarettes, and PMI has been observed using the MRTP decision to lobby for regulatory changes outside of the United States [16]. The use of IQOS’s US market presence in global public relations by PMI also suggests the potential impact of the US import ban outside of the United States. Given the impending return of IQOS to the US market, which may again be used as a global public relations opportunity by PMI, a closer look at how these policy events were received on social media is required to better inform US and global tobacco control policy.

Social media plays a crucial role in analyzing tobacco industry marketing tactics and monitoring discussions on new trends and products, as social media data can provide insights into how general public, news sources, and industry stakeholders react to tobacco control policy changes [17-19]. Further, previous studies highlighted the effectiveness of analyzing Twitter (currently X) data for insights into public’s discourse on tobacco control initiatives [19,20]. Twitter attracts a diverse audience, encompassing various age groups, geographic locations, and interests. Specifically, Twitter was used by 368.4 million people globally in 2022, the time of data collection [21]. In the United States, 23% of adults reported using Twitter, with 42% of its users logging in daily [22,23]. About a quarter of US adolescents between 13 and 17 years old also reported using Twitter [24]. This varied user demographic on Twitter can shed light on a wide array of viewpoints, beliefs, and opinions about HTPs (such as IQOS) after receiving MRTP authorization.

Existing social media studies on IQOS have found that people already saw IQOS as a less harmful alternative to other tobacco products before MRTP authorization [25], with the proportion of tweets with positive attitudes toward IQOS increasing during the month after the MRTP authorization [26]. However, their application in analyzing public reactions to the new policy events that may have a qualitatively different impact on tobacco market in the United States and globally, including the IQOS MRTP authorization in the United States and the US IQOS import ban, remains limited. Therefore, this study adds to the existing literature by collecting new data after new development in HTP-related policies, observing changes in social media discourse during months surrounding significant new policy events that may impact US as well as global tobacco control policies, hence providing further insights. The tobacco industry has been actively using social media for marketing, including promoting IQOS [27,28], warranting close examination of social media content. To understand the potential detrimental impact on perceptions of IQOS and other HTPs, we analyzed HTP-related social media posts during the period surrounding the major policy events described above, analyzing different types of tweets separately with particular attention to user perceptions around health consequences of using HTPs and MRTP-related discussions. This study also adds to existing research by including general keywords related to HTPs, not just IQOS, to capture tweets only using general terms such as “HTP” or “heat-not-burn.”

## Methods

### Data Collection and Procedures

Original Twitter posts (tweets) were collected between June 1, 2020, and December 31, 2021, from Twitter’s Streaming Application Program Interface using IQOS- and HTP-related keywords and hashtags (eg, “iqos,” “heatedtobacco,” and “heatnotburn”; see Section S1 in Multimedia Appendix 1 for the full list). The initial time period for data collection originally was determined immediately before and 1 year after the US MRTP authorization was made in July 2020. During early data collection in fall 2021, a new regulatory event (ruling of IQOS’s patent infringement against BAT’s Glo and subsequent US import ban) emerged, and the research team decided to expand the data collection window to include when the ruling went into effect in November 2021 (see Figure S1 in Multimedia Appendix 1).

Similar to previous studies [25,29], we excluded all retweets to prevent overrepresenting repeated identical statements. Because of limitations in analytical capacity, we limited our analysis to English-language tweets. Geocodes (indicating where the account is located) were included in the metadata, but because people can opt out of including such information, only a small number of tweets included location information, and therefore, geocodes were excluded from analyses.

The initial search yielded 65,333 unique public tweets collected over 19 months; after removing undiscernible or ambiguous content (eg, non-English or emoji-only tweets), we had 10,423 English tweets. We selected a random subset (n=4138) of them (see Figure S1 in Multimedia Appendix 1 for a flow diagram of the tweet selection process) to facilitate manual content analysis.

We developed a codebook informed by prior research [25,26] and refined it during the initial coding and training process (see Tables 1 and 2 for codebook definitions and tweet distribution; see Figure S2 in Multimedia Appendix 1 for more details about the codebook structure and coding procedure). Previous studies coded emerging themes among tweets, including news, health claims, marketing, personal testimonial, policy, or other [25], and examined overall and across-time distributions of neutral-, positive-, or negative-sentiment tweets [26]. Major updates in this codebook include clarifying mutually exclusive categories of tweets (“personal testimonial,” “news/information,” or “direct marketing/retail”) to better reflect the nature of individual tweets and allow separate analyses within each category. We aimed to better focus on personal testimonials that would more closely reflect population perceptions. In addition, 2 main overarching discussion topics, health and policy, were additionally coded irrespective of whether an individual tweet was categorized as personal testimonial, news/information, or direct marketing/retail.

**Table 1:** Distribution of tweets by 3 mutually exclusive categories in the analytic set (n=2796): direct marketing/retail, news/information, and personal testimonials.

Categories^a^			Tweets, n	Corpus (%)	Category (%)	Example
**Personal testimonials: sharing one’s opinions or direct/indirect experiences of HTPs^b^, including perspective from users and nonusers**			1613	57.7	100	Happy that TGA^c^ rejected the selling of heated tobacco products in Australia due to public health and potential harm [URL]
	**Valence**					
		Positive	495	17.7	30.7	I recently bought an IQOS and since then, only smoked 2 cigarettes instead of 30-40. Good cut [URL]
		Neutral	746	26.7	46.2	Watch me buy IQOS after [@] posted it. Always a trigger for me lol
		Negative	372	13.3	23.1	Why are the people that do the IQOS kiosks in Racetrack super friendly? Like you don’t expect someone selling you cancerous products to be as nice as almost all of them are. But they are.
	**Content**					
		Flavor or taste	30	1.1	1.9	[@] I should accept that I couldn’t stop smoking and start vaping. I missed the tobacco taste. Then enters IQOS, now that’s good.
		Experiential remarks	270	9.7	16.7	IQOS smells like farts. How do you smoke that?!
		Information seeking	307	11.0	19.0	[@] Hi, I bought IQOS yesterday and the blade broke. Can you help?
		Comparison to combustible tobacco	401	14.3	24.9	The FDA^d^ has approved #iQOS’ [MRTP^e^] application to claim IQOS is safer than cigarettes, encouraging tobacco companies to make safer products. That’s sound public policy.
		Comparison to ENDS^f^	252	9.0	15.6	#IQOS is a joke. Get a vape or loose leaf vaporizer and avoid being subject to a single brand and its flavors
		Tobacco marketing strategies	198	7.1	12.3	I declined an offer to try IQOS for free by an IQOS salesman. She turned away with a disgusted look #IQOS
**News/information: news articles, statements, or summaries of information and facts about HTPs**			862	30.8	100	New study demonstrates that heated tobacco products caused a notable reduction in Japan for combustible cigarettes sales. Read more: [URL]
	Comparison to combustible tobacco		192	6.9	22.3	EU^g^ countries request #electronic #cigarettes and HTPs to be #taxed like traditional tobacco products [@] #ecigarette #vaping [URL]
	Comparison to ENDS		81	2.9	9.4	New study at Queen Mary University of London shows that IQOS was less effective for reducing cravings since it yielded less nicotine compared to JUUL. [URL] #VapeNews #Stoptober #vape
	Tobacco marketing strategies		113	4.0	13.1	Following video with [@] discusses how the tobacco industry in Guatemala continues using old strategies to promote new products, such e-cigarettes and heated tobacco products. [URL]
	Referring to scientific evidence		225	8.0	26.1	Only 10 of the 62 harmful compounds found in a new IQOS experiment were found in previous studies. Get help for free to quit at [URL] or [Phone number]
**Direct marketing/retail: selling or promoting HTPs through direct marketing claims, including promoting point of sales**			321	11.5	100	[@] New Marlboro IQOS Heat Sticks Black Menthol Flavor, available for international delivery [URL]
	Direct marketing/retail: flavor		66	2.4	20.6	Promotion!!! Taste Amber, Yellow, Turquois, Purple [flavors]. You can pay via Alipay. #iqos #heets #amber #yellow #torquois #purple #heatnoburn #smoke [URL]
	Direct marketing/retail: product features		84	3.0	26.2	Easily eject heat sticks via a sliding button instead of cap removal. Learn more [URL] #london #uk #hnb [URL]

^a^Note: The 3 categories (direct marketing/retail, news/information, and personal testimonials) are mutually exclusive. Within personal testimonials, the 3 valence groups are also mutually exclusive. The remaining content themes within each category are not mutually exclusive. [@], [URL], and [Phone number] indicate redacted information.

^b^HTP: heated tobacco product.

^c^TGA: Therapeutic Goods Administration.

^d^FDA: Food and Drug Administration.

^e^MRTP: modified risk tobacco product.

^f^ENDS: electronic nicotine delivery systems.

^g^EU: European Union.

**Table 2:** Distribution of topics and relevant subthemes in the analytic set (n=2796)

Topics		Tweets, n		Corpus (%)		Topic (%)		Example^a^
**Health**		632		22.6		100		[@] warns that HTP^b^ and traditional cigarettes have similar amounts of nicotine and secondhand aerosol despite being marketed as better alternative
	Comparison to combustible tobacco	298	10.7		47.2		HTP health warnings should reflect their lower risks than cigarettes. Source: [URL] #VapeNews	
	Comparison to ENDS^c^	126	4.5		19.9		[@] Nicotine vapes are undeniably safer than smoking and HNB^d^ products that deliver fewer toxins/carcinogens. Few teens vape. Why is this not seen as a way to quit?	
	Cessation	194	6.9		30.7		[@] I tried IQOS, very good for quitting cigarettes	
**Policy**		809		28.9		100		Altria can’t sell IQOS in US since Biden administration stays out of patent dispute – CNBC: After deadline passed, Altria and PMI unable to sell/import IQOS devices in US [URL] #patentnews
	US-based policy: MRTP^e^	105	3.8		13.0		[@] After FDA^f^ denied ‘modified risk’ order but PM marketed as one, FDA granted an ‘Exposure Modified’ order for #iQOS. No accountability? [@] response to [@] #IQOS order. [URL]	
	US-based policy: Other	450	16.1		55.6		If cigarettes with low nicotine get a PMTA^g^, then all HTP or vaping HTP are bad joke.	
	Non-US policy	244	8.7		30.2		In Japan, e-cigs are illegal, HTPs are legal. Of course HTP lowered cigarette sales. Do not fear. WHO is trying to reverse that by restricting HTP use.	
**HTP brand mentioned**		2796		100		100		
	IQOS	1838	65.7		65.7		My JUUL and IQOS witness my daily pack of cedars blue cigarettes [URL]	
	Other brands	205	7.3		_7.3_		[URL] [@] will release new heated tobacco product Ploom X on Aug 17 at convenience stores in Japan and certain retail stores. Currently available for presale	
	General HTPs	835	29.9		_29.9_		Hong Kong Council on Smoking and Health advise the banning of HNB and e-cigarettes due to misleading lower health risks. These risks have been proven globally [URL]	

^a^Note: [@], [URL], and [Phone number] indicate redacted information.

^b^HTP: heated tobacco product.

^c^ENDS: electronic nicotine delivery systems.

^d^HNB: heat-not-burn.

^e^MRTP: modified risk tobacco product.

^f^FDA: Food and Drug Administration.

^g^PMTA: premarket tobacco product authorization.

Tweets were categorized into four mutually exclusive categories: (1) personal testimonials, (2) news/information, (3) direct marketing/retail, or (4) noise (irrelevant or not understandable). We focused our analysis on the 2796 tweets that fit into categories 1-3.

The unit of analysis was the text of the tweet; images were not included in the analyses. When coding the tweets, we only considered information that was explicitly mentioned—that is, content that may have been included in the linked websites but not discussed in the tweets was not considered. Also, many tweets include a long list of hashtags at the end, with many hashtags only loosely related to the actual tweet content or adding any new information. Therefore, we only considered hashtags that were part of the actual tweet sentences (eg, hashtags considered: “Globally, #HTPs help millions of smokers and are a safer alternative”; not considered: “HTPs: next generation of smoke-free alternatives for teens #heatedtobacco #tobacco #prevention”) [30].

The tweets within each category were coded into specific subthemes (not mutually exclusive), using similar approaches from previous research [25,26] as well as via iterative discussion during the coder meetings. Among personal testimonials, six subthemes emerged: (1-a) description of flavor or taste in the context of a personal recommendation, thoughts, or review; (1-b) personal experience using HTPs either favorably or unfavorably (eg, feeling of smoke and taste); (1-c) information seeking about HTPs (eg, troubleshooting and asking about product availability); (1-d) comparison to combustible tobacco; (1-e) comparison to electronic nicotine delivery systems (ENDS); or (1-f) HTP marketing strategies. Personal testimonial tweets were also coded for their valence: positive, neutral, or negative. News/information tweets were coded for four subthemes: (2-a) comparison to combustible tobacco, (2-b) comparison to ENDS, (2-c) tobacco marketing strategies, or (2-d) referring to scientific evidence. Direct marketing/retail tweets were coded for two subthemes: (3-a) flavors and (3-b) product features.

All tweets were further coded for 2 not mutually exclusive topics: health (mentioning health risks and/or benefits of using HTPs; subthemes included comparison to combustible tobacco, comparison to ENDS, and/or cessation) and/or policy (discussing HTP-related tobacco policy or regulation; subthemes included mentions of MRTP authorization, other US-based policy, and/or policy outside of the United States). Tweets were also coded for specific HTP brands (not mutually exclusive): whether they mentioned IQOS, other HTP brands (eg, Ploom and Glo), or referred to general product category.

Three independent coders coded the tweets using a binary system (yes/no) for each category, topic, and subtheme (see Figure S2 in Multimedia Appendix 1). To establish interrater reliability, 3 coders double-coded a subsample of tweets (total 300, approximately 10% of the analytic sample [19,30,31]) through an iterative procedure, where differences across coders in the initial practice set were discussed in real time and coding definitions were amended and refined. Coders applied the refined codebook to the next smaller set and repeated this process until consensus was reached and inter-rater agreement reached at least 90%. Because of a lack of variance for some codes (ie, too many 0’s vs 1’s), κ could not be reliability calculated; therefore, we used percent agreement instead to assess inter-rater reliability. The first author served as an arbitrator resolving discrepancies between the coders. Then the 3 coders each independently coded a third of the remaining tweets.

### Data Analysis

After completing the coding process, we conducted a descriptive analysis of the tweets according to categories and themes. We also performed a pairwise co-occurrence analysis to assess connections across themes: for example, we examined the proportion of personal testimonials tweets that also discussed health-related content about HTPs. To visualize the co-occurrence matrix containing the counts of co-occurrences of themes, the *igraph* package version 1.2.6 in R (R Foundation for Statistical Computing) was used [25]. Examining the co-occurrence of themes, separate analyses were conducted within the mutually exclusive categories, and we present results from the personal testimonials and news/information tweets, excluding direct marketing/retail tweets that were the least frequent.

### Ethical Considerations

As this study analyzed publicly accessible information only, no ethics board review has been conducted. All URLs and individual Twitter handles were redacted (eg, “[URL]” rather than reproducing what was included in the tweet) to protect privacy. To further protect privacy, posts used in this paper were paraphrased by one of the coders so that the tweets included cannot be easily traced back to original accounts.

## Results

### Distribution of Tweets Across Time

**Figure 1:**
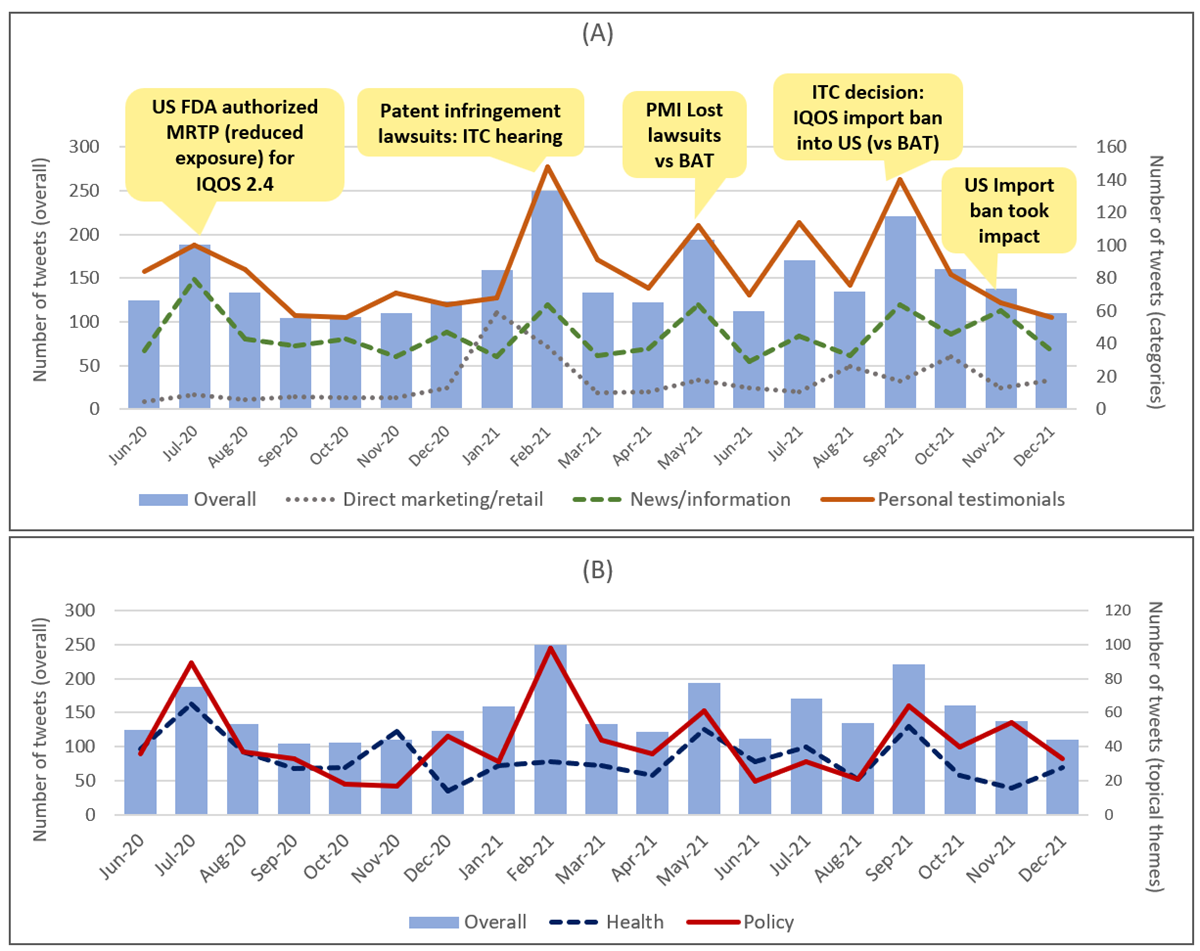
Distribution of tweets (n=2976) between June 2020 and December 2021 and relevant policy events. (A) Overall tweets (left axis) and 3 categories (right axis: direct marketing/retail, news/information, and personal testimonials). (B) Overall tweets (left axis) and 2 topical themes (right axis: health and policy). BAT: British American Tobacco; FDA: Food and Drug Administration; ITC: International Trade Commission; MRTP: modified-risk tobacco product; PMI: Philip Morris International.

The distribution of tweets across the 19-month period is shown in Figure 1, along with several policy and business events. Compared with June 2020, a 50% increase in the number of tweets was observed in July 2020 after the IQOS MRTP authorization. Other peaks in the number of tweets were observed in February, May, and September 2021, related to events surrounding IQOS patent infringement lawsuits and the US import ban. This trend was mostly driven by news/information and policy-related tweets. Health-related tweets were most frequent in July 2020, immediately after the US FDA’s MRTP authorization of IQOS reduced exposure claims. Personal testimonials were most frequent in February and September 2021, coinciding with legal disputes and the US import ban. Direct marketing*/*retail tweets were generally lower in number, with a peak observed in January 2021.

### Categories and Topical Themes

#### Descriptive Characteristics

Tables 1 and 2 show the descriptive characteristics of tweets and paraphrased example tweets illustrating each category (Table 1: mutually exclusive) and topic (Table 2: not mutually exclusive) with relevant subthemes. Overall, the most prevalent category was personal testimonials (1613/2976, 57.7%), followed by news/information (862/2976, 30.8%), and direct marketing/retail (321/2976, 11.5%). In terms of topic, policy (809/2976, 28.9%) was more prevalent than health (632/2976, 22.6%).

#### Categories of Tweets: Personal Testimonials, News/Information, and Direct Retail/Marketing

Within personal testimonials, close to half of the posts (746/1613, 46.2%) were neutral, followed by positive (495/1613, 30.7%) and negative (372/1613, 23.1%) posts. Examples of positive tweets often included mentions of reduced harm (or its potential), for example, “For the past year, IQOS has been great for my lungs and throat” or discreet nature of using HTPs (eg, “I like how IQOS makes smoking discreet. I can smoke so much at home without anyone noticing.”). Some tweets simply expressed favorable attitudes toward IQOS and other HTPs, such as “Falling in love with my IQOS, more every day.” Examples of negative tweets included suspicion toward the tobacco industry (eg, “#PhillipMorris views menthol #cigarette ban as opportunity to market heated tobacco products and make money, not harm reduction. Stop regulatory loopholes. Heated tobacco is tobacco.”), or the perception that HTPs do not differ from smoking cigarettes (eg, “IQOS is same as smoking with many extra hassle, including charging and higher cost. Terrible.”).

Personal testimonial tweets comparing HTPs to combustible tobacco appeared more frequently (401/1613, 24.9%) than those comparing HTPs to ENDS (252/1613, 15.6%), with 8.1% (131/1613) mentioning both comparisons. In both comparisons, positive was the most prevalent sentiment (199/401, 49.6%, for combustible and 117/252, 46.4%, for ENDS comparisons), followed by neutral (119/401, 29.7%; 78/252, 31.0%) and then negative toward HTPs (83/401, 20.7%; 57/252, 22.6%). Positive comparisons of HTPs to combustible tobacco testimonials generally pointed to harm reduction potential and lack of smoke, whereas comparisons to ENDS were mixed. Some mentioned that HTPs and vaping are “on the same side” in terms of harm reduction, whereas some mentioned differential preference between vaping and HTPs (eg, “ecigs just doesn't work for everyone. IQOS and snus are much more satisfying but heated tobacco is not everyone's cup of tea. Respect diverse tastes.”). Among negative tweets, comparisons to both combustible tobacco and ENDS often discussed that HTPs are similar to (or no better than) smoking (eg, “Researchers compared IQOS and cigarettes but weren't able to find enough data to show they are different on cardiovascular problems in testing over short time.”).

Nearly half (407/862, 47.2%) of news/information tweets discussed policy (see the “Topical Themes: Health and Policy” section below), including the IQOS MRTP authorization (eg, “FDA authorizes IQOS marketing with reduced exposure claims [URL]”). Tweets in this category often compared HTPs to combustible tobacco (192/862, 22.3%) or ENDS (81/862, 9.4%), most of which mentioned the potential health risks and benefits of HTPs (eg, “PMI claimed compared to cigarettes, IQOS aerosol includes 95% fewer toxins. However, this and other similar results all come from tobacco industry-funded research.”). About a quarter of news/information tweets (225/862, 26.1%) cited scientific evidence, such as peer-reviewed studies or other data sources (eg, “An #onlinefirst study showed that heated tobacco products, e-cigarettes, and cigarettes are correlated with biomarkers of stiff arteries.”).

Only 11.5% (321/2796) of all tweets belonged to this category, mostly referring to online retailers and accompanied by web links. Among these, 20.6% (66/321) mentioned flavors (eg, “Mellow tobacco blend and zesty aroma notes in Yellow Selection HEETS tobacco sticks for IQOS that are now available for purchase”). Although none of these tweets included geocodes, we noted that 45.2% (145/321) of the direct marketing/retail tweets included phone numbers, region names, or currency units, with the United Arab Emirates being the most frequent (92/321, 28.7%; eg, “IQOS HEETS Sienna available in Dubai/Abu Dhabi”), followed by the United Kingdom (22/321, 6.9%; eg, “Come visit our stores to learn about Ploom from in-store experts. We are always here to help you! #Ploom #PloomUK #HeatedTobacco #Westfield”). A total of 9.7% (31/321) of marketing/retail tweets mentioned “international delivery” as an available option.

#### Topical Themes: Health and Policy

Nearly a quarter (632/2796, 22.6%) of the tweets were health related. Similar to what we observed among personal testimonials, about half of health-related tweets (298/632, 47.2%) compared HTPs with combustible tobacco, whereas only 19.9% (126/632) compared HTPs with ENDS. More than a quarter (194/632, 30.7%) of health-related tweets discussed IQOS’s efficacy for cigarette cessation, approximately three-quarters of which included one’s own experience using IQOS either successfully (eg, “Try IQOS. I've quit traditional cigs for a year and it's awesome!”) or unsuccessfully (eg, “I was a smoker for 12 years. I tried to stop smoking by using IQOS, but I failed… now I need to quit both”). Only 9 direct marketing/retail tweets discussed health (eg, “If you want to switch from cigarettes, give Ploom S Kit a try. Order yours from the [Shop name]”).

More than a quarter (809/2796, 28.9%) of all tweets discussed policy: the majority of them (450/809, 55.6%) discussed US-based policy other than MRTP authorization, such as marketing authorization under premarket tobacco product application for IQOS 2.4 (April 2020) or IQOS 3 (December 2020), or IQOS import ban to the United States (September 2021). Discussion of non-US policy was also a common topic among policy tweets (244/809, 30.2%; eg, “New German tobacco law states tax increase of 3% on cigarettes, 26% on HTPs, and 1000% on e-cigs”). MRTP authorization (105/809, 13.0%) discussions were relatively uncommon.

Although the FDA made it clear that their MRTP marketing order for IQOS is limited to reduced exposure, not reduced risks, some tweets claimed that this decision proves that IQOS is safer than other tobacco products and referred to the authorization decision as FDA “approval” (eg, “Yes they are safe - FDA approved them because they see IQOS as appropriate for #publichealth protection.”). Some tweets discussed that this US policy will also impact global marketing of IQOS (eg, “…RJ Reynolds won the lawsuit against PMI, so IQOS cannot be sold in the US. But their FDA approval stays and will help PMI to promote IQOS globally.”).

### Thematic Co-occurrences

**Figure 2:**
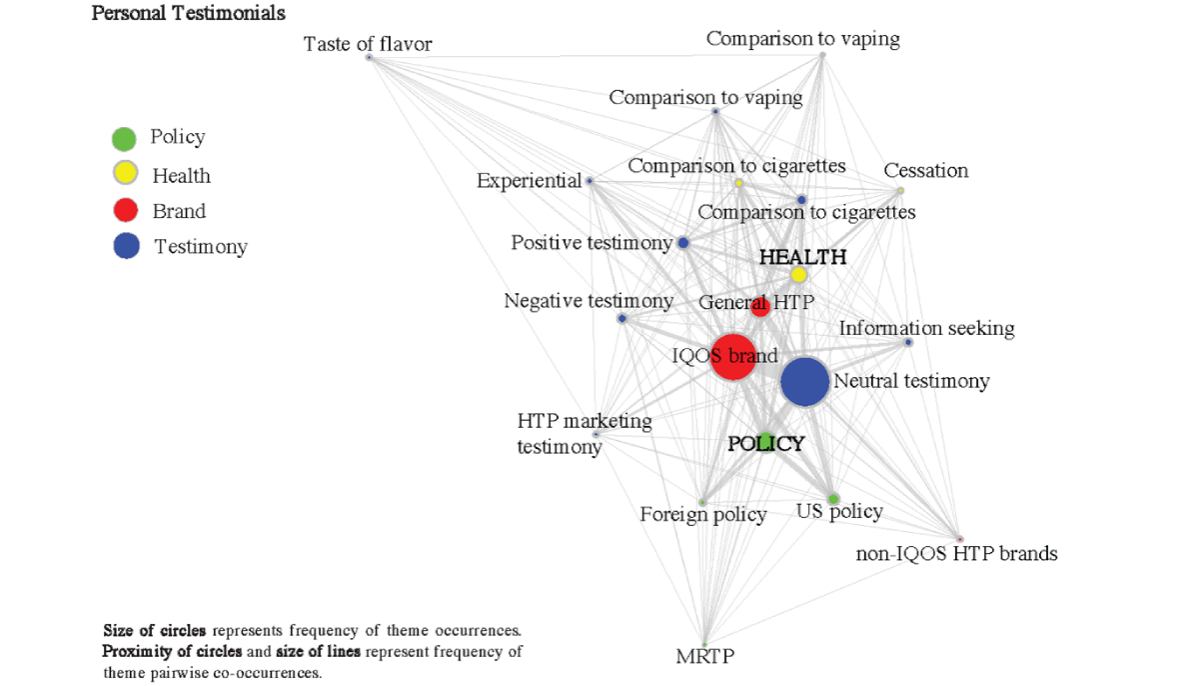
Theme co-occurrences of personal testimonials (n=1613) with policy (n=399), health (n=445), and brand (IQOS: n=1173; other: n=82; general HTPs: n=424). HTP: heated tobacco product.

**Figure 3:**
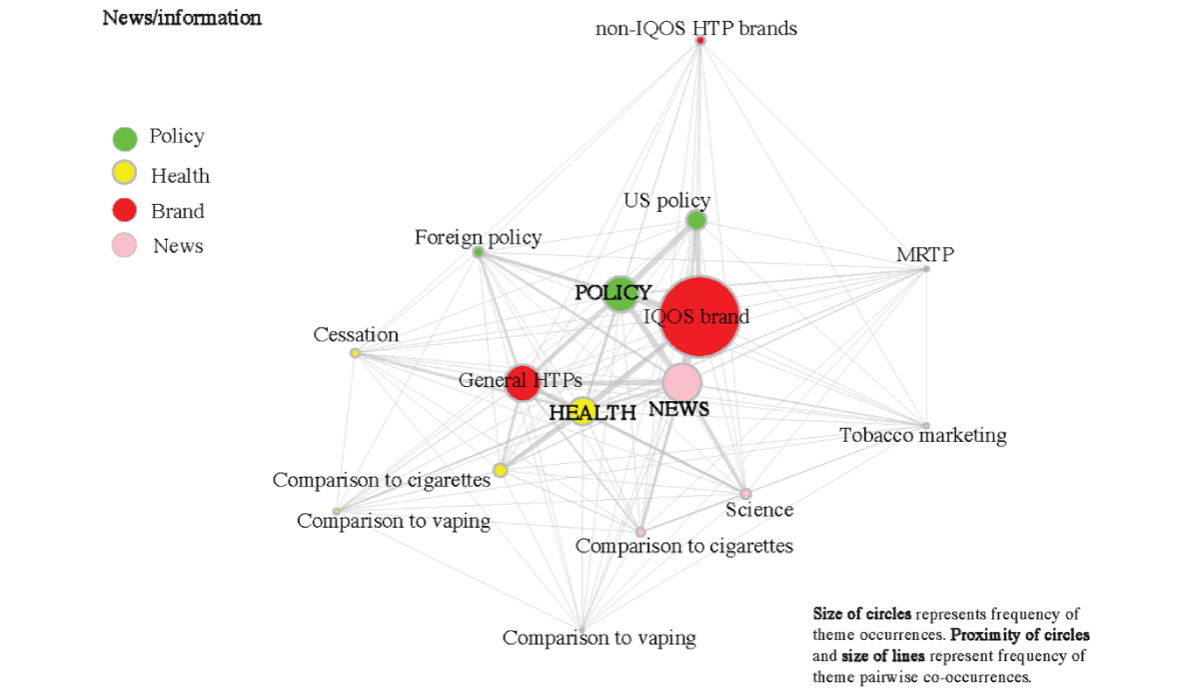
Theme co-occurrences of news/information (n=862) with policy (n=407), health (n=278), and brand (IQOS: n=447; other: n=30; general HTPs: n=393). HTP: heated tobacco product; MRTP: modified-risk tobacco product.

Figures 2 and 3 display separate co-occurrence analyses within personal testimonial (Figure 2) and news/information (Figure 3) tweets. Among personal testimonials, neutral tweets mentioning IQOS brand (634/1613, 39.3%) showed the largest co-occurrence, followed by neutral tweets discussing policy (378/1613, 23.4%) and neutral tweets about general HTPs (268/1613, 16.6%). There were slightly more health-related positive testimonials (220/1613, 13.6%) than health-related negative testimonials (144/1613, 8.9%). News/information tweets had the highest co-occurrence with IQOS brand (447/862, 51.9%), policy (407/862, 47.2%), general HTPs (393/862, 45.6%), followed by scientific evidence (225/862, 26.1%), US-based policy (223/862, 25.9%), and health (178/862, 20.6%). The thickness of the lines represents the frequency of pairwise co-occurrences, and the size of the circles represents the frequency of theme occurrences.

## Discussion

### Principal Findings

This study reviewed tweets related to HTPs between June 2020 (1 month before the US FDA’s MRTP authorization for IQOS 2.4) and December 2021 (1 month after the IQOS US import ban took effect) to observe people’s response to major policy decisions related to HTPs. Social media such as Twitter, Reddit, and TikTok have emerged as a major source of news and other information, especially among young people [32,33]. Tobacco industry has also been actively using social media as a promotion outlet for emerging tobacco products [27,28]. A recent study on Instagram suggests that despite the platform’s attempts at regulating tobacco promotions, paid partnership posts are abound promoting venues and events that feature tobacco use, suggesting limited compliance and/or enforcement [34]. Social media use is associated with greater exposure to tobacco marketing and promotions [35] and more favorable perceptions toward and use of nicotine and tobacco products [36,37]. Coupled with concerns related to the lack of regulations and enforcement due to their global nature and strong reliance on user-created content [38,39], a closer surveillance is warranted for social media discourse surrounding emerging tobacco products.

We found that more than half of the tweets were personal testimonials, expressing thoughts and opinions based on one’s own experience and/or knowledge, which provide a valuable window into public opinion regarding HTPs. Close to half of the personal testimonials were neutral, either not expressing clear valence or mentioning both positive and negative perspectives. Among the non-neutral personal testimonials, consistent with a previous study [25], more tweets expressed a positive than negative valence toward HTPs. In positive-valence tweets, people described IQOS as less harmful, “better,” or “safer” than combustible tobacco products, namely cigarettes, or discussed HTPs as being able to help people who smoke quit smoking. Another study examining IQOS-related tweets between November 2019 and August 2020 (2 months after MRTP authorization) found that overall there were more negative than positive tweets, but also found that positive tweets were more frequently observed around MRTP authorization [26]. This study suggests that this trend continued during the year after MRTP authorization. This is also consistent with PMI’s harm reduction marketing strategies in and outside of the United States [3,40,41], including their global public relations efforts emphasizing the US FDA’s MRTP authorization [16,42], and may reflect explicit and implicit marketing influence. However, it should be noted that the US FDA’s MRTP authorization for IQOS was limited to reduced exposure to harmful chemicals and did not include reduced risks of tobacco-related disease, compared to cigarettes [7]. With insufficient evidence on whether or how much IQOS or other emerging tobacco products may reduce the health risks in the short and long term compared to cigarettes [43], such discussions should be closely monitored and, if necessary, corrected using effective communication campaigns.

### Comparison With Prior Work

This study provides recent examination of social media discourse by collecting recent dataset from Twitter. This is also extremely timely given the impending return of IQOS to the US market after a legal settlement between PMI and BAT, which will resolve ongoing litigations [10]. Compared to an early study [25], tweets that directly market or promote HTPs comprised a substantially lower proportion of the corpus (Barker et al [25]: 32.3%; this study: 11.5%). The observed reduction in direct marketing may be a result of an increase in other categories. For example, personal testimonials may have increased as IQOS is known to more people after MRTP authorization and PMI’s continued promotional effort globally. Or, this may reflect the tobacco industry’s shift to more stealthy marketing where paid influencers post promotional content as personal opinion, as the tobacco industry has been historically doing [28]. In addition, our timeline involved multiple major policy events surrounding IQOS. There may have been more policy-related discussions in both the personal testimonial and news/information categories demonstrated by our co-occurrence analysis, resulting in a relative decrease in the proportion of direct marketing/retail tweets. This finding may also reflect relatively lower direct marketing/retail activities on Twitter than other social media platforms, such as Instagram and YouTube, by IQOS official accounts [27].

Direct marketing/retail tweets were observed most often during January 2021. This could be because the HTP retailers may target those who want to stop smoking combustible cigarettes as part of their new year’s resolutions, suggesting switching to HTPs. Such cessation claims have been observed in early marketing and promotion of e-cigarettes [44-46]. However, there were no specific health or cessation claims observed in direct marketing/retail tweets in our analytic set. Most tweets surrounding cessation claims were personal testimonials, in which individual Twitter users discussed their own experience of smoking cessation using HTPs.

It should be noted that HTP marketing is regulated by many social media platforms [39]. Moreover, the US FDA’s MRTP marketing order requires that all digital marketing for IQOS be allowed only with audience age verification and mandates continuous monitoring and reporting of all promotional activities [47]. However, content on social media and other online channels that originates overseas can easily reach an international audience, including people in the United States. Our search, conducted from the United States, included multiple direct marketing/retail tweets that could be seen without an age verification. Content of some direct marketing/retail tweets suggested that many originated from outside of the United States, such as from the United Arab Emirates or the United Kingdom, with some tweets offering “international delivery.” Therefore, despite the US regulation that restricts shipping of tobacco products [48], it should be closely examined whether retailers operating from overseas may be able to circumvent national retail and promotional restrictions [49].

This study adds to existing information on social media discussions about other emerging tobacco products such as ENDS [29,30,50] by examining how an emerging product (ie, HTPs) is discussed in comparison to ENDS and cigarettes. Our findings are consistent with existing research that people who post on Twitter may misjudge the emerging products’ relative risks to other tobacco products [50], as some posts we reviewed suggested confusion between MRTP authorization and approval, and included frequent mentions of reduced health risks compared to cigarettes. This study also adds to existing social media content analyses of HTPs [25,26] by including tweets from more recent time points that overlap with many meaningful policy events (eg, MRTP authorization and US import ban). Although these events’ direct legal impact is limited to the United States, PMI has been using MRTP authorization in their global public relations efforts [42] and lobbying for international tobacco regulatory policy changes [16]. Moreover, a news content analysis in low- and middle-income countries found that half of the 50 identified news articles covering US MRTP authorization during the 6 months after the announcement included misleading “reduced risk” language [51] even though the US FDA’s decision only specifies “reduced exposure” to harmful and potentially harmful chemicals. This may lead to incorrect risk perceptions around IQOS and other HTPs in and outside of the United States and calls for the needs of close surveillance of public opinion and effective health communication campaigns to inform appropriate regulatory policies and interventions globally.

### Limitations

This study also comes with limitations that are not uncommon in similar studies examining social media content. First, this study only considered posts from publicly accessible accounts and may not reflect the attitudes of people with private Twitter accounts. Additionally, because of the analytical capacity, we limited our corpus to English language only. This potentially misses much conversation happening in non–English speaking countries such as Japan, Germany, and Italy, where IQOS seems to have gained greater popularity [52], as well as conversations conducted in non-English languages commonly spoken in the United States (eg, Spanish). Previous research suggests that Twitter users’ information-sharing networks largely cluster and segregate as a function of language and topic [53], and Spanish speakers in the United States may be more likely to receive and share online misinformation in general [54]. However, it is important to note that a recent analysis of Spanish tobacco-related tweets found similar themes to previous English-only studies [55].

Some of the personal testimonials may have come from influencers connected to and sponsored by the tobacco industry, without clear disclosure within the tweet [28,56]. We did not investigate the nature of the accounts posting tweets—if the text reflected a person’s direct or indirect experience and/or opinion toward IQOS, we counted it as a personal testimonial. A similar approach was applied to news/information tweets, where tweets from tobacco industry public relations accounts may have inadvertently been included. A future study should explore the origins of such tweets and how the differences across account types may differentially impact viewers’ perceptions.

The location of tweets (eg, US vs non-US) would have been helpful to better understand the impact of the US-based policy events on global tobacco control policy; however, because of the small number of tweets (approximately 2%) that include location information, such analyses could not be conducted. Future content analyses may provide further insights if they can verify information where the posts originated.

Moreover, our analyses focused on the textual content of tweets, not including external links or images. This may have led to some loss of information. For example, some tweets were vague about which authorization they referred to (eg, “Senate plenary to deliberate regulation of import, manufacturing, sales, distribution, and use of vapes and HTPs”), so it was unclear whether they should be coded as US or foreign policy. It is also possible that some additional themes and subthemes would have emerged if we included images in the coding process [57]. This study also collected data only from Twitter from a specific time period, and therefore the results may not generalize to other popular social media platforms such as Facebook, Instagram, or TikTok that have different format features, platform regulations, and user populations.

### Conclusions

Our study suggests that social media content analysis can be a useful tool for public opinion surveillance related to emerging tobacco products, including the sentiment of social media users regarding emerging tobacco products. Such information should be actively used to determine what kind of interventions and communication campaigns should be prioritized. This study categorized and analyzed 3 different types of tweets discussing HTPs: personal testimonials, news/information, and direct sales/marketing. Our findings suggest that after the US FDA’s IQOS MRTP authorization, many Twitter users held neutral or positive perspectives toward HTPs. They perceived IQOS as being less harmful or safer than cigarettes, even though the MRTP authorization was limited to “reduced exposure” claims, not “reduced risk.” Given that many people turn to social media as a source of information, examining news/information tweets also provides insight into the nature of information about HTPs that people are exposed to. This includes some areas for concern, including misrepresentation of MRTP authorization as “FDA approval.” This study also raises awareness about cross-border marketing and sales of HTPs and calls for stricter regulatory policy and effective enforcement on social media platforms globally. This kind of content analysis provides more detailed information driven by the themes of users’ posts and a different perspective from traditional surveys, although continuous research is required to address its representativeness.

## Appendix

Multimedia Appendix 1 Search keywords for relevant tweets, selection process of the analytic set, and coding procedure and category/content theme and subtheme structure.

## Abbreviations

BAT: British American Tobacco
ENDS: electronic nicotine delivery system
FDA: Food and Drug Administration
HTP: heated tobacco product
ITC: International Trade Commission
MRTP: modified-risk tobacco product
PMI: Philip Morris International

## Data Availability

Analytic set and coding results may be shared with reasonable request.
