# Supplementary material for: Discussion of Heated Tobacco Products on Twitter Following IQOS’s Modified-Risk Tobacco Product Authorization and US Import Ban: Content Analysis
Source: J Med Internet Res. 2024 Oct 24;26:e53938. doi: 10.2196/53938 (PMC11544331; doi:10.2196/53938)

**Multimedia Appendix 1**

**Section S1. Search keywords for relevant tweets**

Widely used terms related to heated tobacco products (e.g., heat not burn), their acronyms (e.g., HNB, HTP), and major brand names from PMI (e.g., IQOS, HeatSticks, Heets) and other companies (e.g., Ploom) were included. “Glo” (BAT’s HTP product) was initially included but later excluded from search terms as it yielded too much noise.

Final keyword list: *"iqos*", "heatnotburn", "heatedtobacco", "Heet*", "Heatstick*", "tobaccoheatingsystem", "ploom", “htp”, “hnb”*

Covered timeline: 6/1/2020 – 12/31/2021

**Figure S1. Selection process of the analytic set (n=2796)**


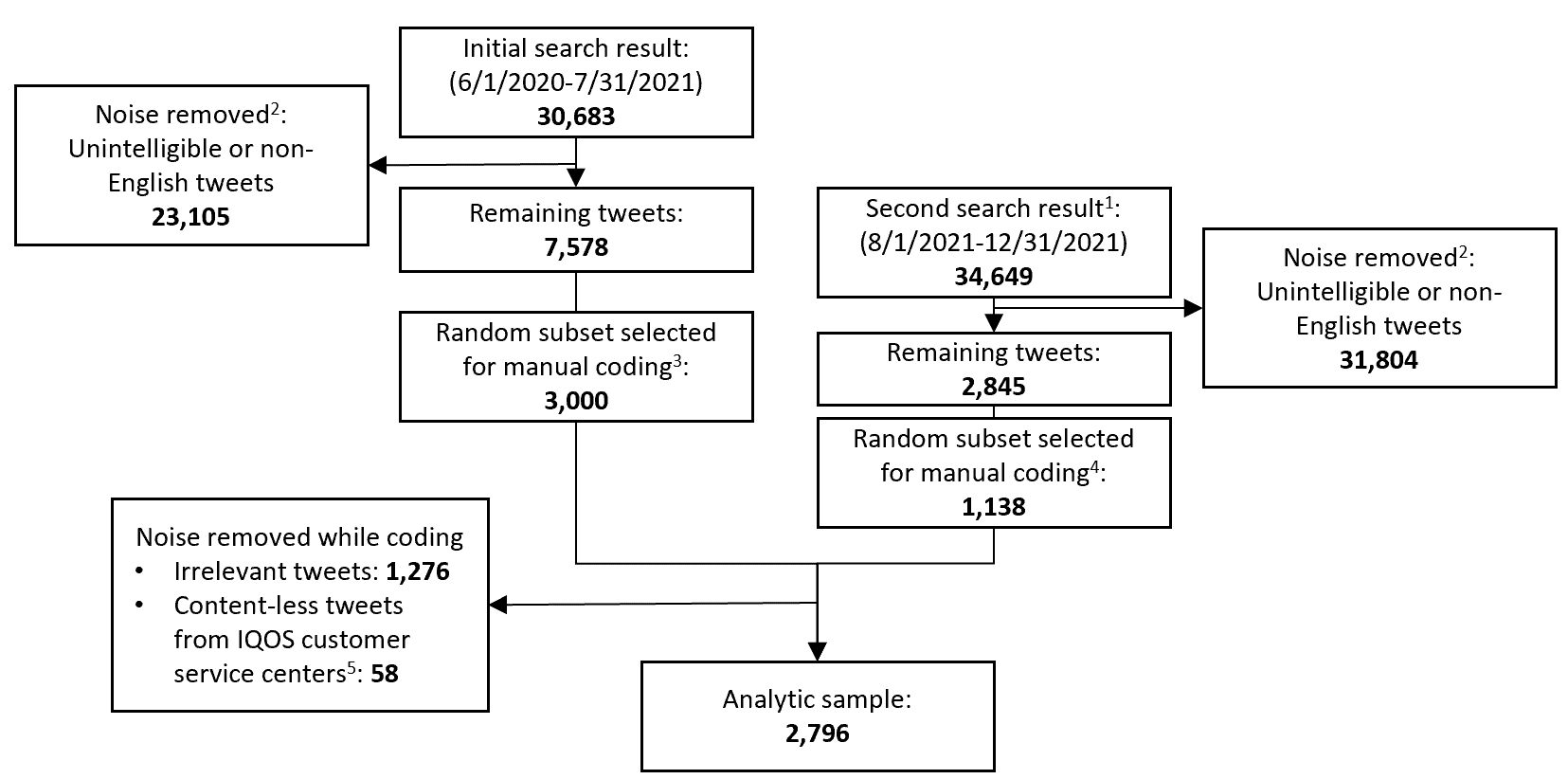


*Note*. 1. We acquired additional set of tweets using the same set of search terms has been conducted as new regulatory event (Ruling of IQOS’s patent infringement against RJ Reynolds/BAT’s Glo) happened during the analysis.
2. Non-English tweets and noise (irrelevant words, e.g., “sheets” erroneously tagged with the search term “heets”) were removed from the initial and the second search results. The second search yielded more non-English tweets than the first set, resulting in smaller proportion of remaining tweets.
3. We confirmed that the proportion of tweets in each month does not differ between random subset and overall search result.
4. The proportion of random subset was maintained from initial search set.
5. These were all same, copy-and-pasted tweets from IQOS customer service mentioning those who seek help with IQOS. e.g., *“[@] We are UK IQOS support account. Please DM us with your date of birth to confirm you are 18+ and a smoker/IQOS user.**”*

**Figure S2. Coding procedure and category/content theme and subtheme structure**


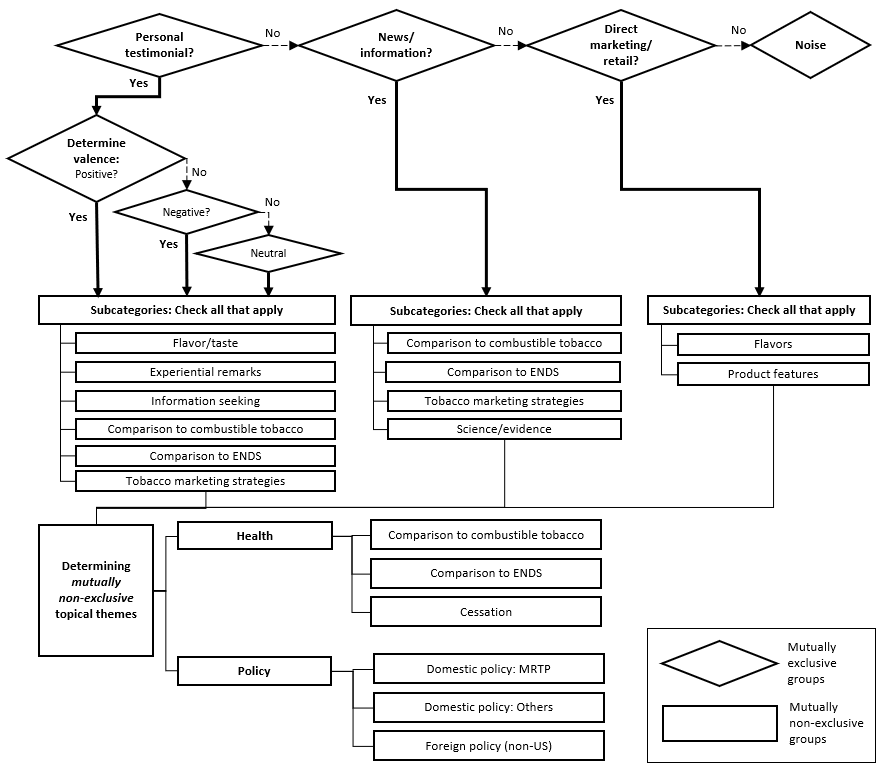

Supplement: Multimedia Appendix 1 [file jmir_v26i1e53938_app1.docx]
